# Supplementary material for: Sex chromosome and sex locus characterization in goldfish, Carassius auratus (Linnaeus, 1758)
Source: BMC Genomics. 2020 Aug 11;21:552. doi: 10.1186/s12864-020-06959-3 (PMC7430817; doi:10.1186/s12864-020-06959-3)
Supplement: Supplementary file 1 — Additional file 1 Table S1. Sequences of the primers used for Y-allele genotyping in goldfish. [file 12864_2020_6959_MOESM1_ESM.docx]

**Table S1. Sequences of the primers used for Y-allele genotyping in goldfish.**

| **Primers** | | **PCR product (bp)** | **Genome location** | |
| --- | --- | --- | --- | --- |
| **names** | **Sequence(5’ - 3’)** |  | Male assembly | NCBI_genome |
| Marker 1 | Forward: AATACAACATTCCCAGGGAGTGCA  Reverse: CATCAAGGGCTATCTGACCAAGA | 1169 | Flattened_line_394560:620-1788 | NW_020523543.1 |
| Marker 2 | Forward: GTGCTCAATAGACGACGGATTCTC  Reverse: GTCTGTCTGTTAGCCTGTTCTCCA | 1189 | Flattened_line_270798:2006-3194 | NW_020525535.1 |
| Marker 3 | Forward: GATGAAGGTCTCGGTCTGTTGTTA  Reverse: CCCTGTTATGTTTGTATTGGCTAC | 2548 | Flattened_line_35862:4409-6956 | NC_039250.1 (LG8) |
| Positive control | Forward: AAGAGCGCCTCCTAGTGTTT  Reverse: GAGACGGAGGAGTGGTATCG | 994 | Flattened_line_0:6858-7842 | NC_039245.1 (LG3) |

Three Y-allele primer pairs (marker 1 to 3) and one autosomal primer pair (positive control) were designed on our XY male genome assembly (male assembly). Name of the contig and nucleotide position (3’-5’) are given in the genome location column with the corresponding National Center for Biotechnology Information Accession numbers.
