# Supplementary figures and images for: Sex chromosome and sex locus characterization in goldfish, Carassius auratus (Linnaeus, 1758)
Source: BMC Genomics. 2020 Aug 11;21:552. doi: 10.1186/s12864-020-06959-3 (PMC7430817; doi:10.1186/s12864-020-06959-3)

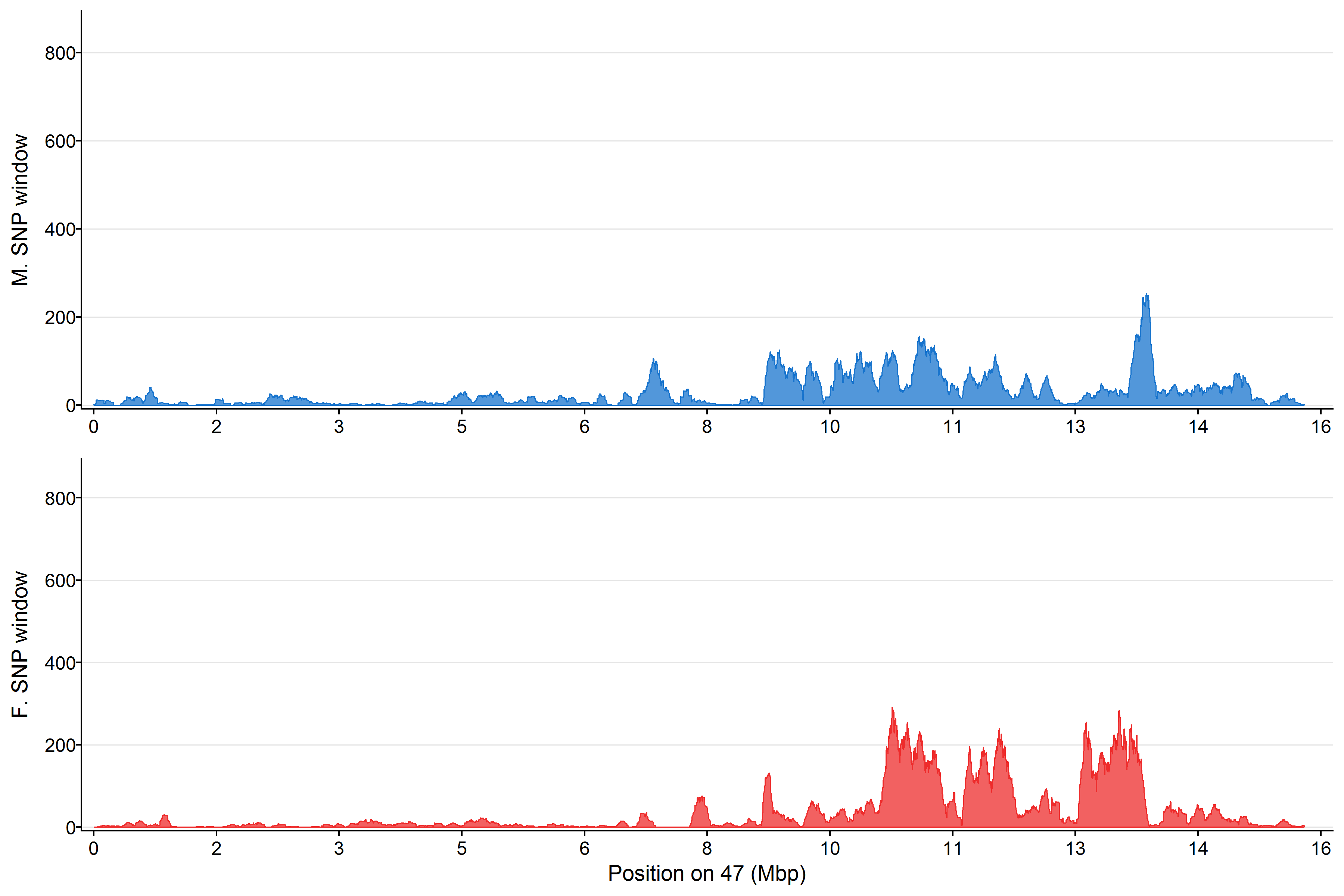

Supplement: Supplementary file 2 — Additional file 2 Figure S1. Distribution of sex-biased SNPs on LG47. SNPs were counted using 100 kb sliding window with an output point every 500 bp. The top panel displays the profile of male-specific SNPs (blue area), while the bottom panel displays the profile of female-specific SNPs (red area). [file 12864_2020_6959_MOESM2_ESM.png]

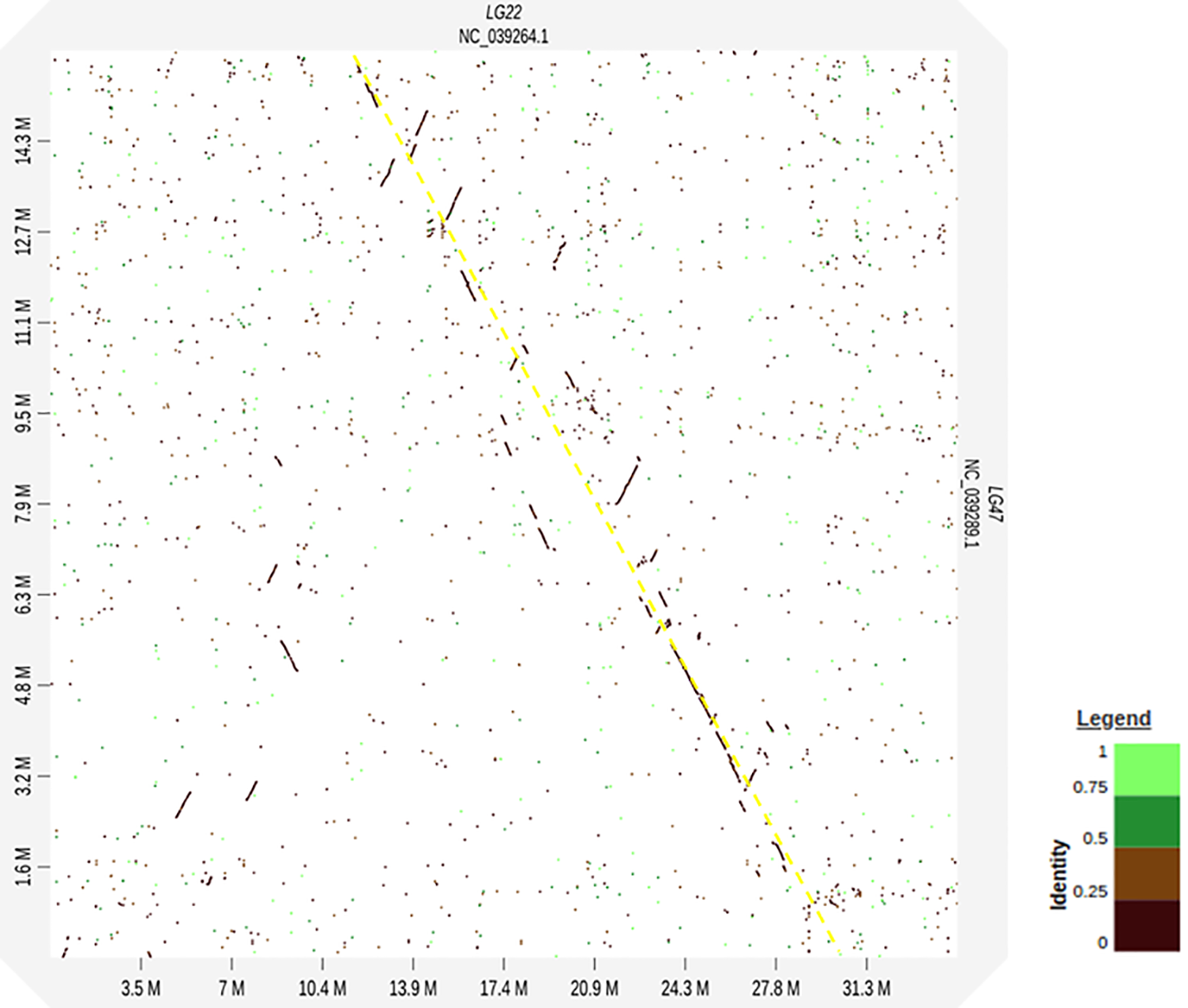

Supplement: Supplementary file 3 — Additional file 3 Figure S2: Dot plot comparison of LG22 and LG47 showing conserved synteny between these two linkage groups. [file 12864_2020_6959_MOESM3_ESM.png]

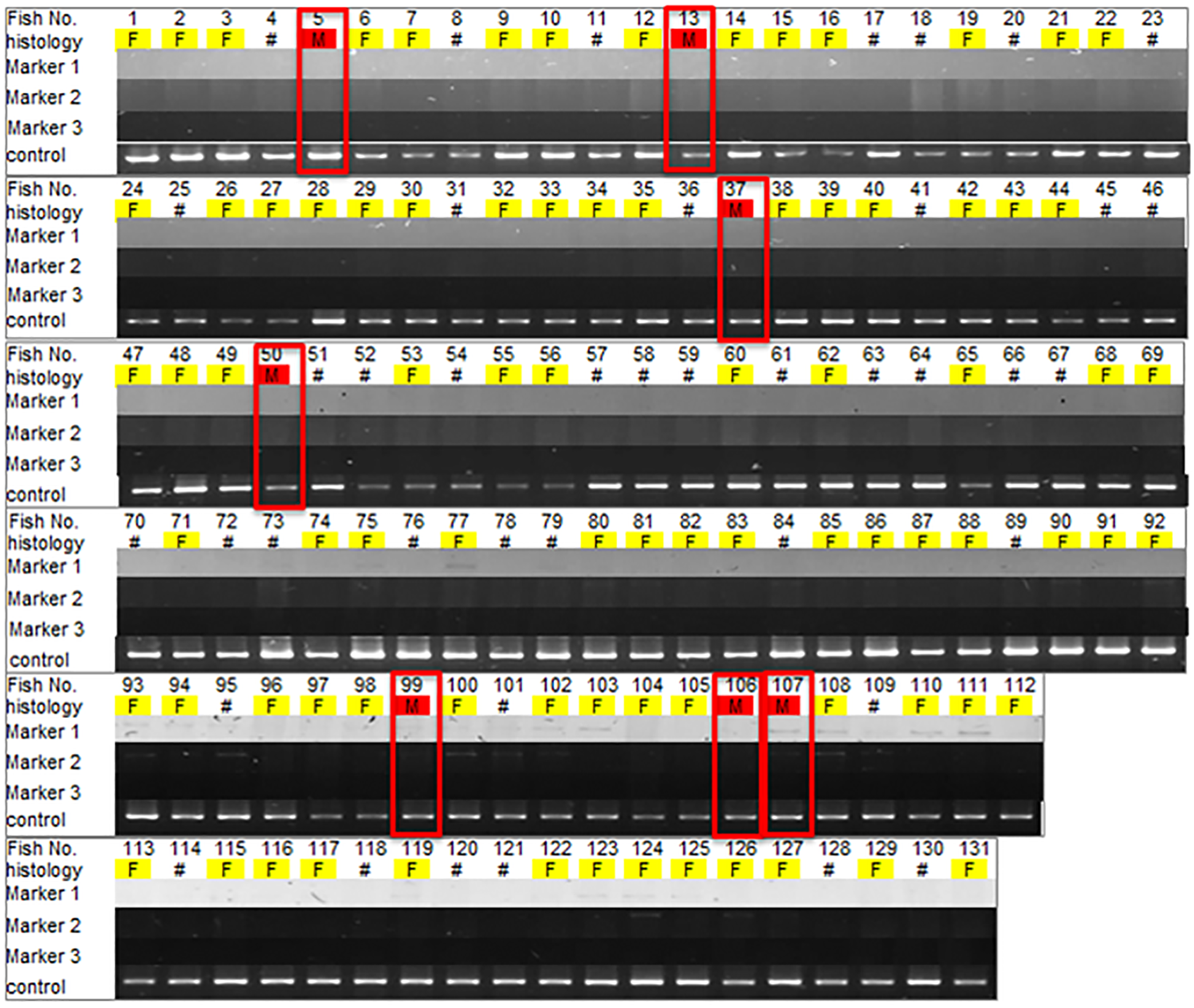

Supplement: Supplementary file 4 — Additional file 4 Figure S3: Sex genotyping with Y-allele primers of the offspring of a putative XX neomale with a normal XX female. Genotyping was conducted with three Y-allele primers and one autosomal primer used as a gDNA quality control. Phenotypic sex was determined by gonadal histology and males and females are shown using red and yellow color respectively. Female-to-male sex-reversed animals (N = 7) are highlighted by red boxes. Hashes indicate animals with unknown phenotypic sex with undifferentiated gonads based on histology. The original, unprocessed gel images of this figure are available in additional file 5. [file 12864_2020_6959_MOESM4_ESM.png]

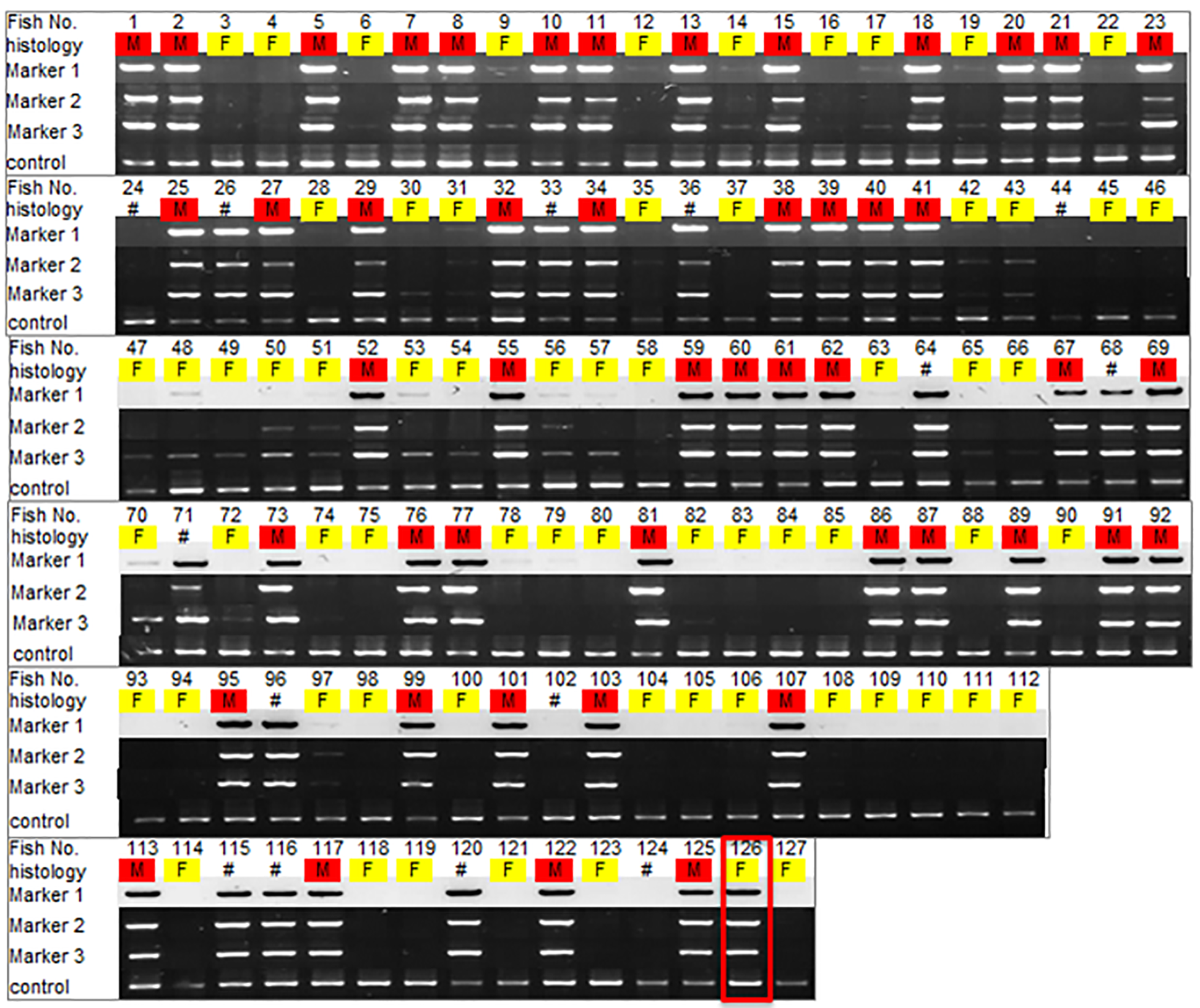

Supplement: Supplementary file 5 — Additional file 5 Figure S4. Sex genotyping with Y-allele primers of the offspring of a putative XY male with a normal XX female. Genotyping was conducted with three Y-allele primers and one autosomal primer used as a gDNA quality control. Phenotypic sex was determined by gonadal histology and males and females are shown using red and yellow color respectively. The female-to-male sex-reversed animal (N = 1) is highlighted by a red box. Hashes indicate animals with unknown phenotypic sex with undifferentiated gonads based on histology. The original, unprocessed gel images of this figure are available in additional file 5. [file 12864_2020_6959_MOESM5_ESM.png]
