# Supplementary material for: Sex chromosome and sex locus characterization in goldfish, Carassius auratus (Linnaeus, 1758)
Source: BMC Genomics. 2020 Aug 11;21:552. doi: 10.1186/s12864-020-06959-3 (PMC7430817; doi:10.1186/s12864-020-06959-3)
Supplement: Supplementary file 10 — Additional file 10. Original, unprocessed gel images of Fig. 1b, and supplementary figures 3 and 4. [file 12864_2020_6959_MOESM10_ESM.docx]

**Supplementary materials to:**

**Sex chromosome and sex locus characterization in goldfish, *Carassius auratus* (Linnaeus, 1758)**.

Ming Wen^1,2^, Romain Feron^2,3,4^, Qiaowei Pan^2,3^, Justine Guguin^2^, Elodie Jouanno^2^, Amaury Herpin^2^, Christophe Klopp^5,6^, Cedric Cabau^6^, Margot Zahm^6^, Hugues Parrinello^7^, Laurent Journot^7^, Shawn M. Burgess^8^, Yoshihiro Omori^9,10^, John H. Postlethwait^11^, Manfred Schartl^12^, Yann Guiguen^2^*

* Correspondance: Yann Guiguen: [yann.guiguen@inrae.fr](mailto:yann.guiguen@inrae.fr)

**AFFILIATIONS:**

^1^ State Key Laboratory of Developmental Biology of Freshwater Fish, College of Life Science, Hunan Normal University, Changsha, China

^2^ INRAE, LPGP, 35000, Rennes, France.

^3^ Department of Ecology and Evolution, University of Lausanne, 1015 Lausanne, Switzerland.

^4^ Swiss Institute of Bioinformatics, 1015 Lausanne, Switzerland.

^5^ Plate-forme bio-informatique Genotoul, Mathématiques et Informatique Appliquées de Toulouse, INRAE, Castanet Tolosan, France.

^6^ SIGENAE, GenPhySE, Université de Toulouse, INRAE, ENVT, Castanet Tolosan, France.

^7^ Montpellier GenomiX (MGX), c/o Institut de Génomique Fonctionnelle, 141 rue de la Cardonille, 34094, Montpellier Cedex 05, France.

^8^ Translational and Functional Genomics Branch, National Human Genome Research Institute, Bethesda, MD, USA.

^9^ Laboratory of Functional Genomics, Graduate School of Bioscience, Nagahama Institute of Bioscience and Technology, Nagahama, Shiga, Japan.

^10^ Laboratory for Molecular and Developmental Biology, Institute for Protein Research, Osaka University, Suita, Osaka, Japan.

^11^ Institute of Neuroscience, University of Oregon, Eugene, Oregon, USA.

^12^ Developmental Biochemistry, Biozentrum, University of Würzburg, Würzburg, Germany and The Xiphophorus Genetic Stock Center, Department of Chemistry and Biochemistry, Texas State University, San Marcos, Texas, USA.

1. **Original argarose gel images of Figure 1.B**


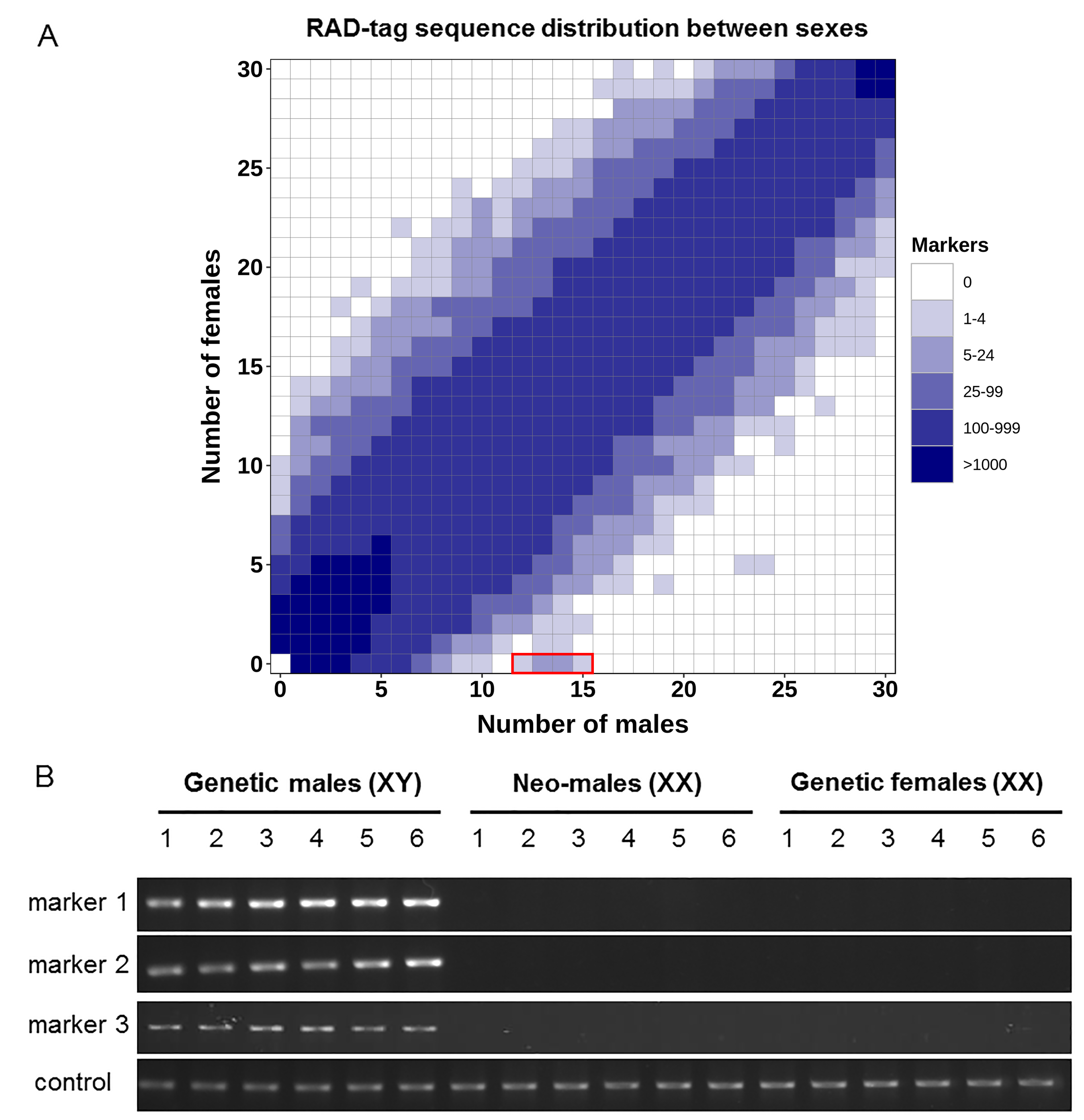


Original gel figure for marker 1, marker 2 and control:


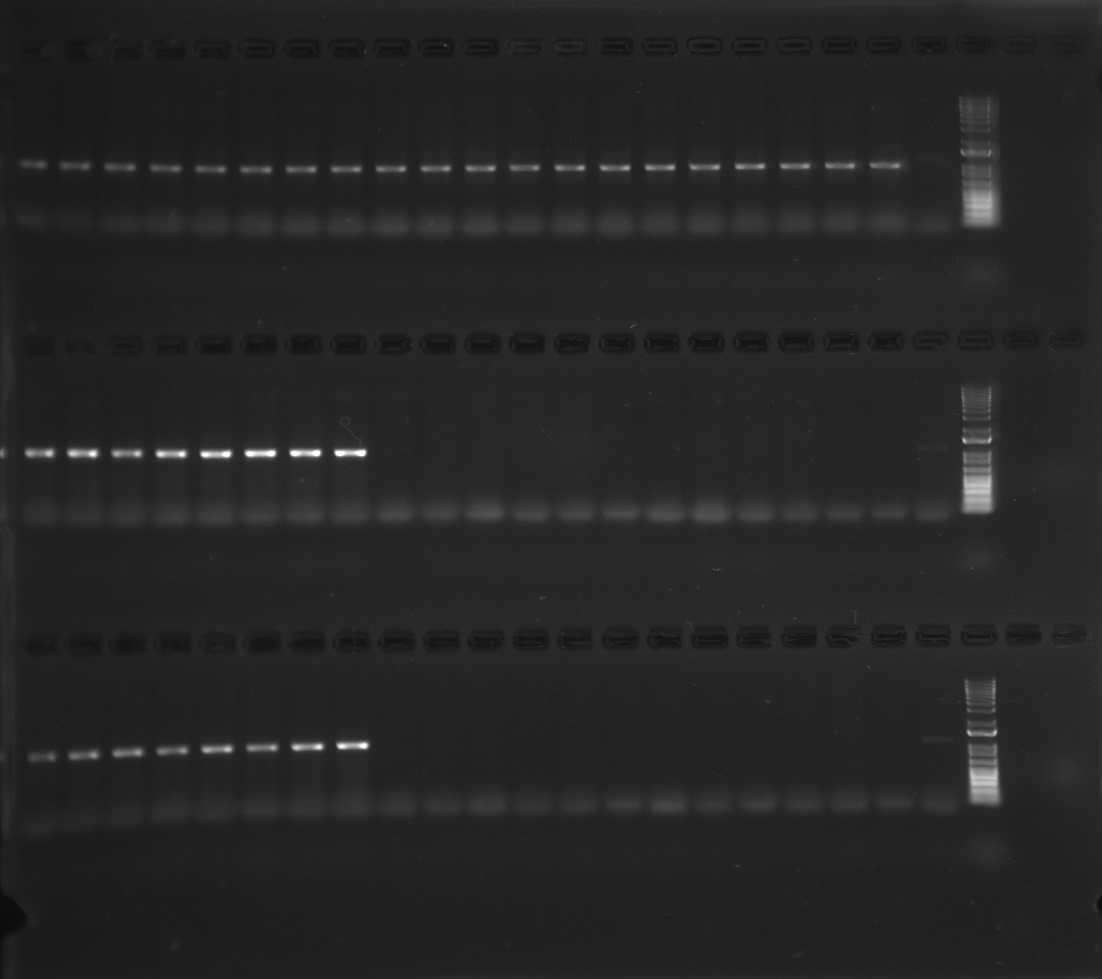


Genetic males (XY)

Genetic males (XY)

Genetic males (XY)

2

3

4

5

6

1

2

3

4

5

6

1

2

3

4

5

6

1

1

6

5

4

3

2

1

6

5

4

3

2

1

6

5

4

3

2

Genetic males (XY)

Genetic males (XY)

Genetic males (XY)

Genetic males (XY)

Genetic males (XY)

Genetic males (XY)

1

6

5

4

3

2

1

6

5

4

3

2

6

1

2

5

4

3

Marker 2

Marker 1

Control

Original gel figure for marker 3:


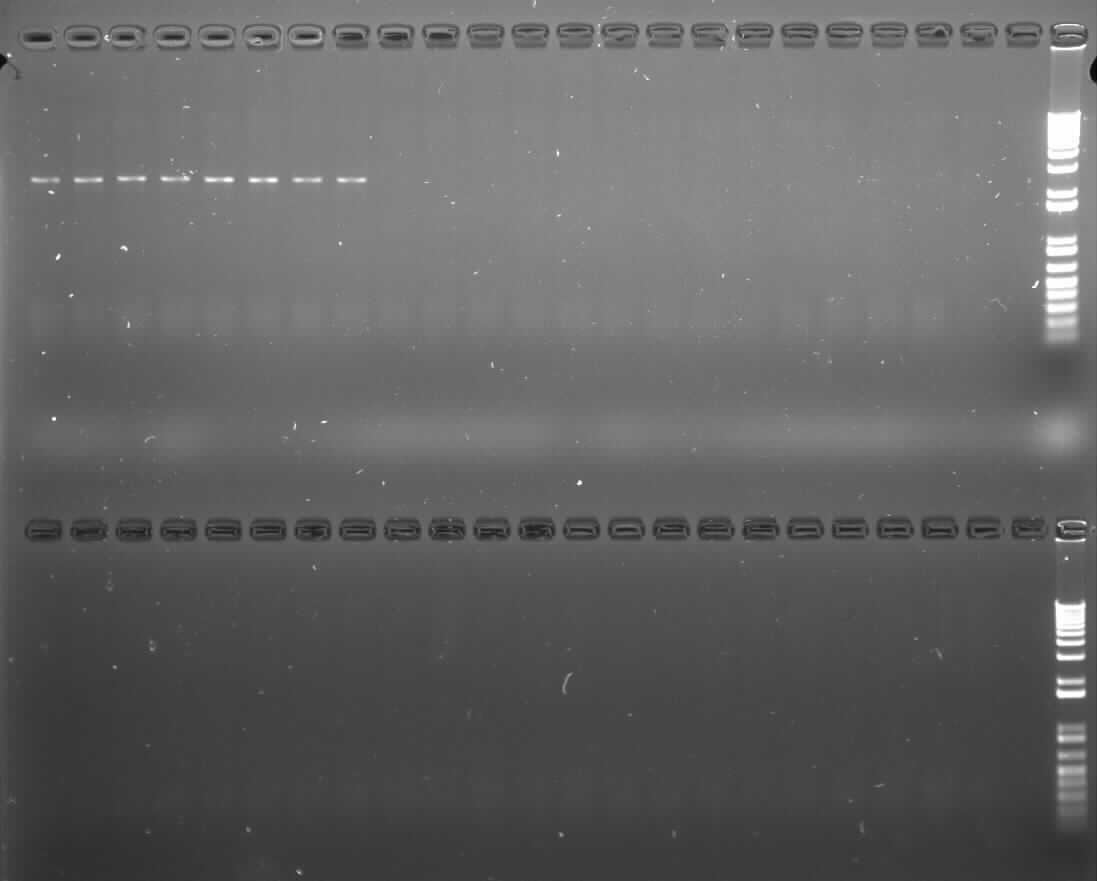


Genetic males (XY)

Genetic males (XY)

Genetic males (XY)

2

3

4

5

6

1

2

3

4

5

6

1

2

3

4

5

6

1

Marker 3

**Figure 1B | Final figure and original gel images of figure 1B.** The final figure is shown on the top with the original gel images below with the cropped regions shown as a white box.

1. **Original gel images of Figure S3**


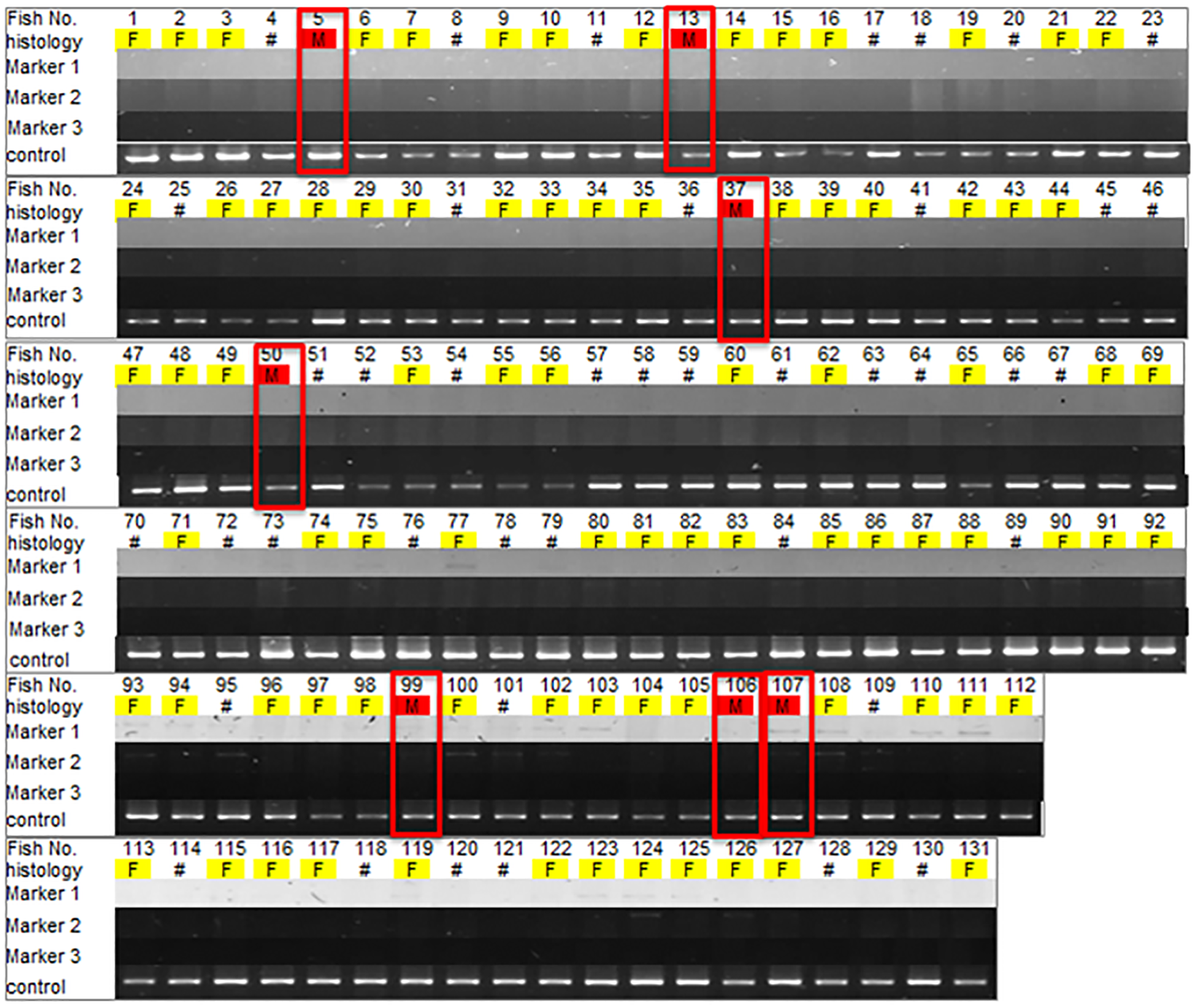


Original gel figure for marker 1:


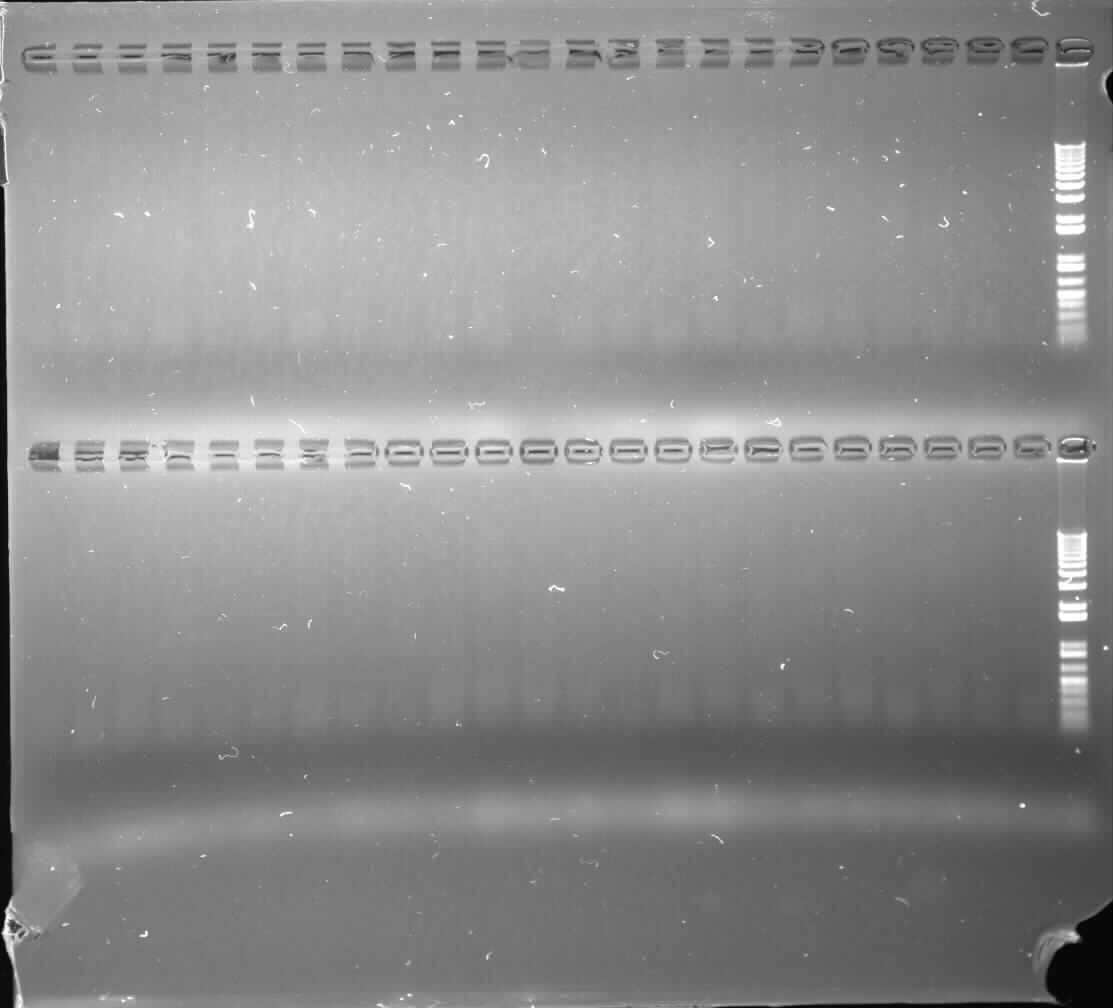


Marker 1: 24 - 46

Marker 1: 1 - 23


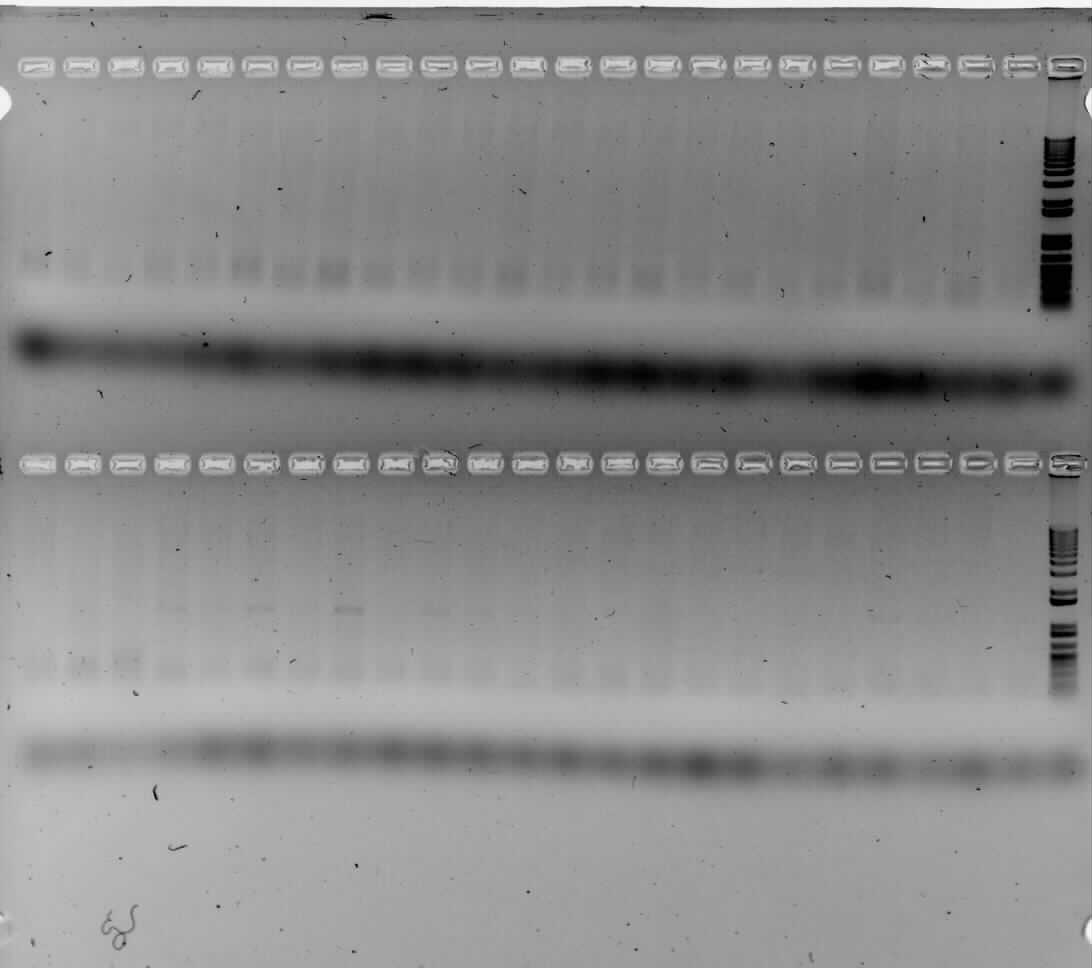


Marker 1: 70 - 92

Marker 1: 47 - 69


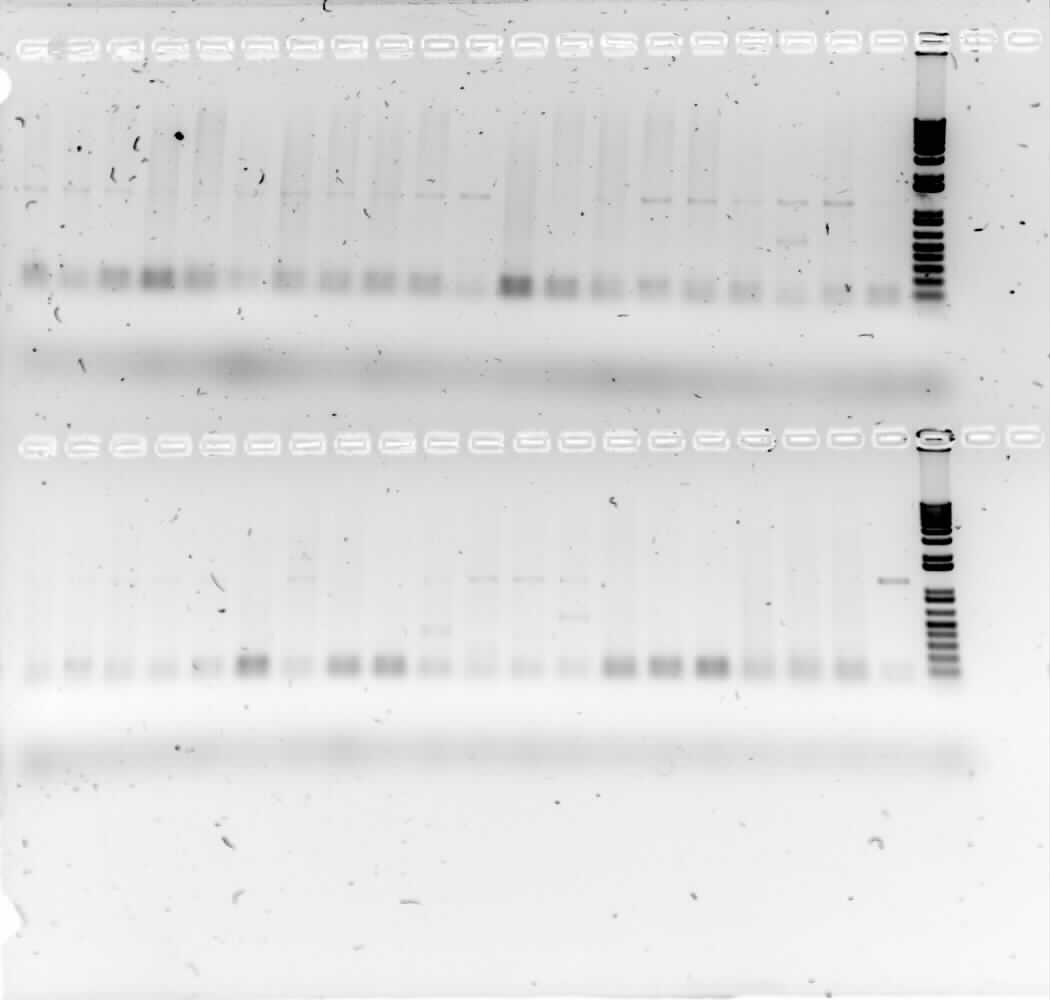


Marker 1: 113 - 131

Marker 1: 93 - 112

Original gel figure for marker 2:


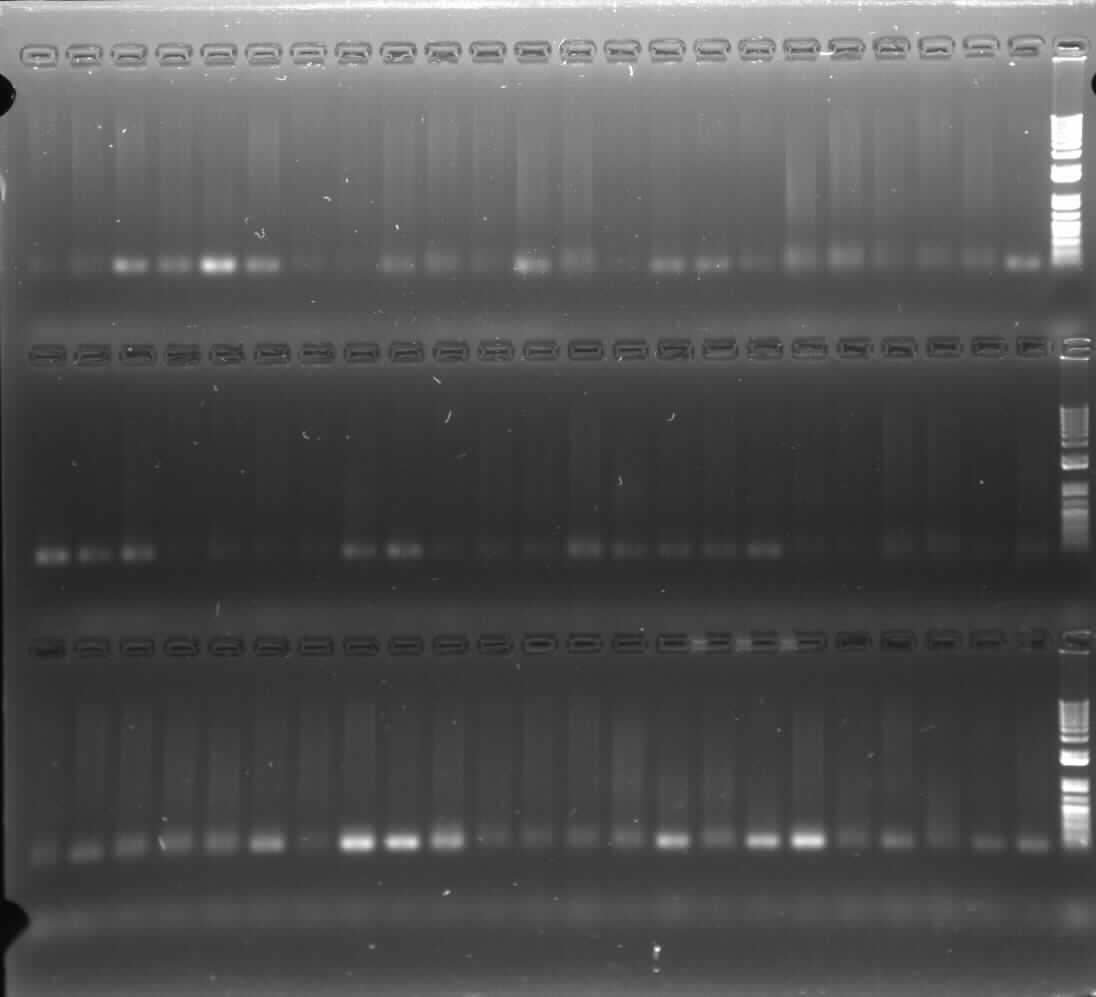


Marker 2: 47 - 69

Marker 2: 24 - 46

Marker 2: 1 - 23


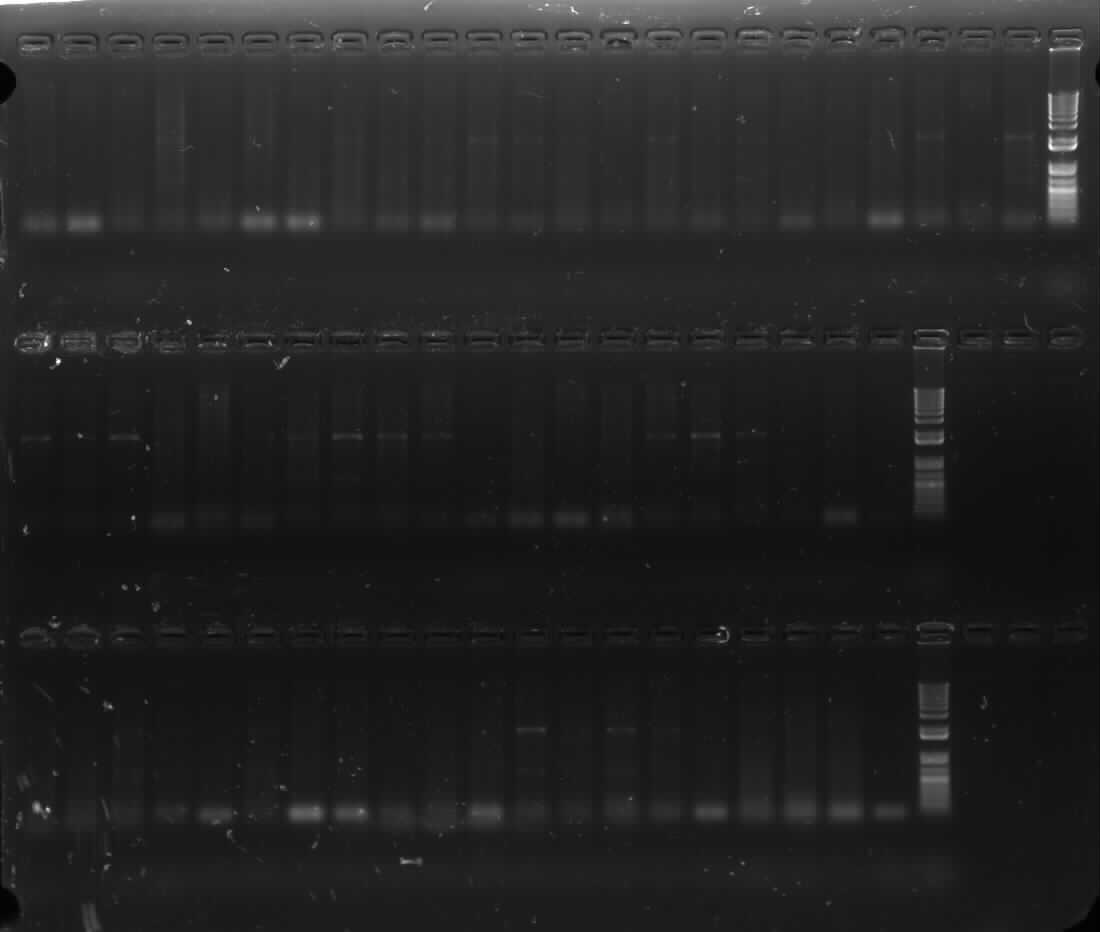


Marker 2: 113 - 131

Marker 2: 93 - 112

Marker 2: 70 - 92

Original gel figure for marker 3:


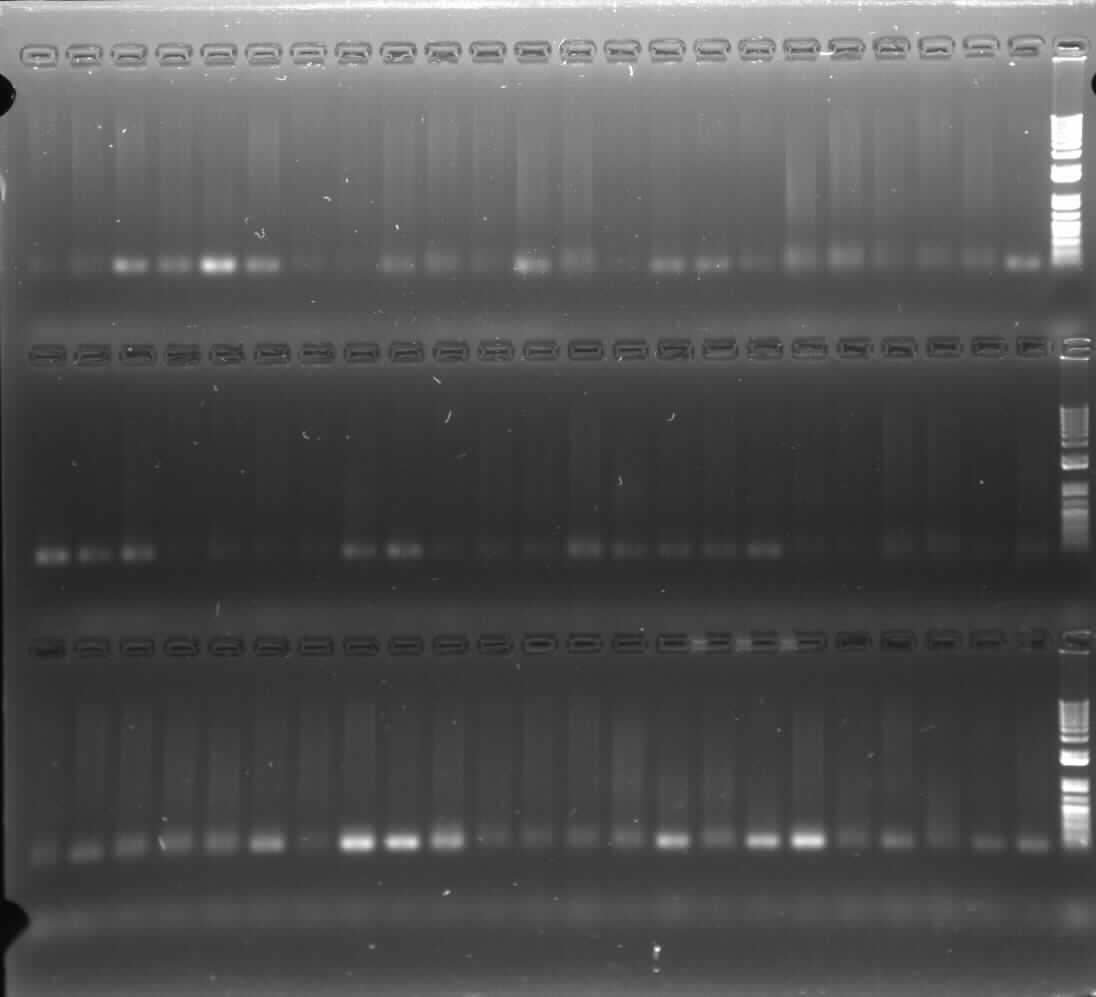


Marker 3: 47 - 69

Marker 3: 24 - 46

Marker 3: 1 - 23


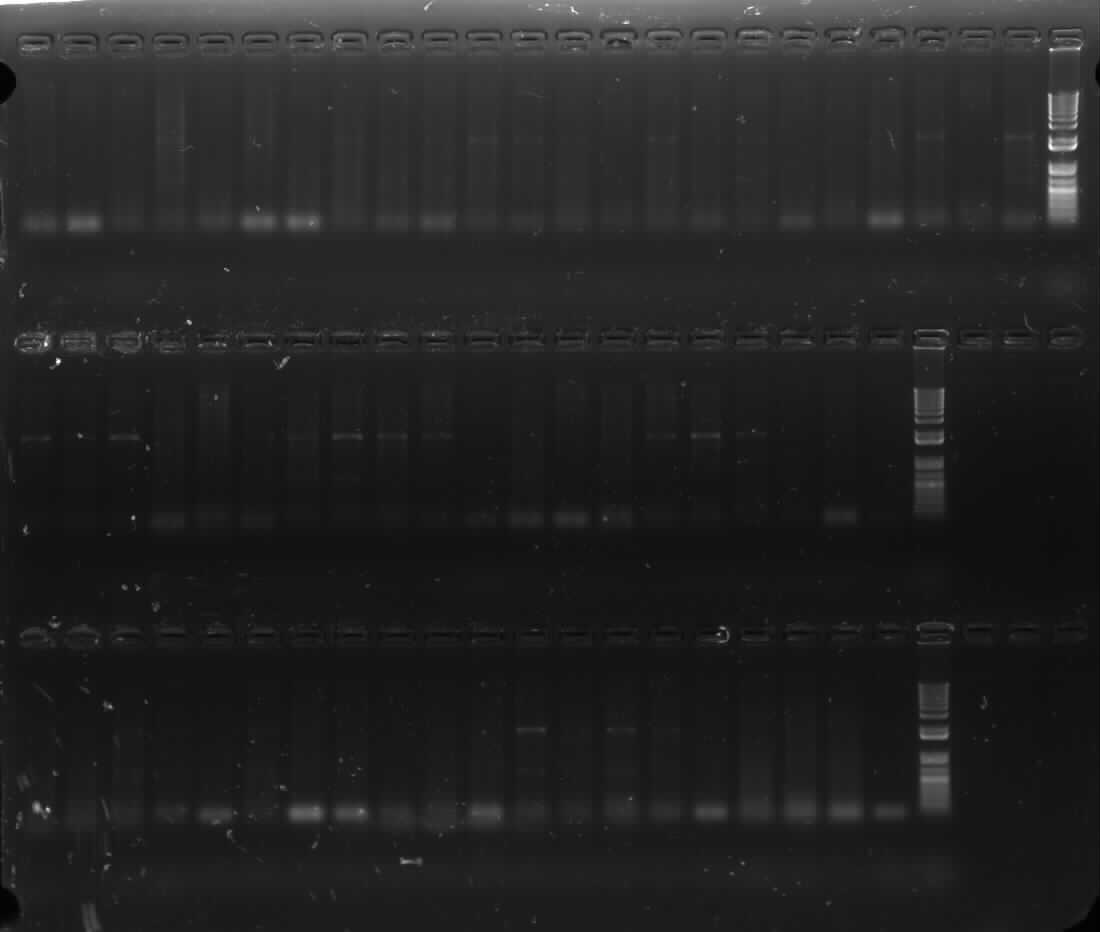


Marker 3: 113 - 131

Marker 3: 93 - 112

Marker 3: 70 - 92

Original gel figure for control:


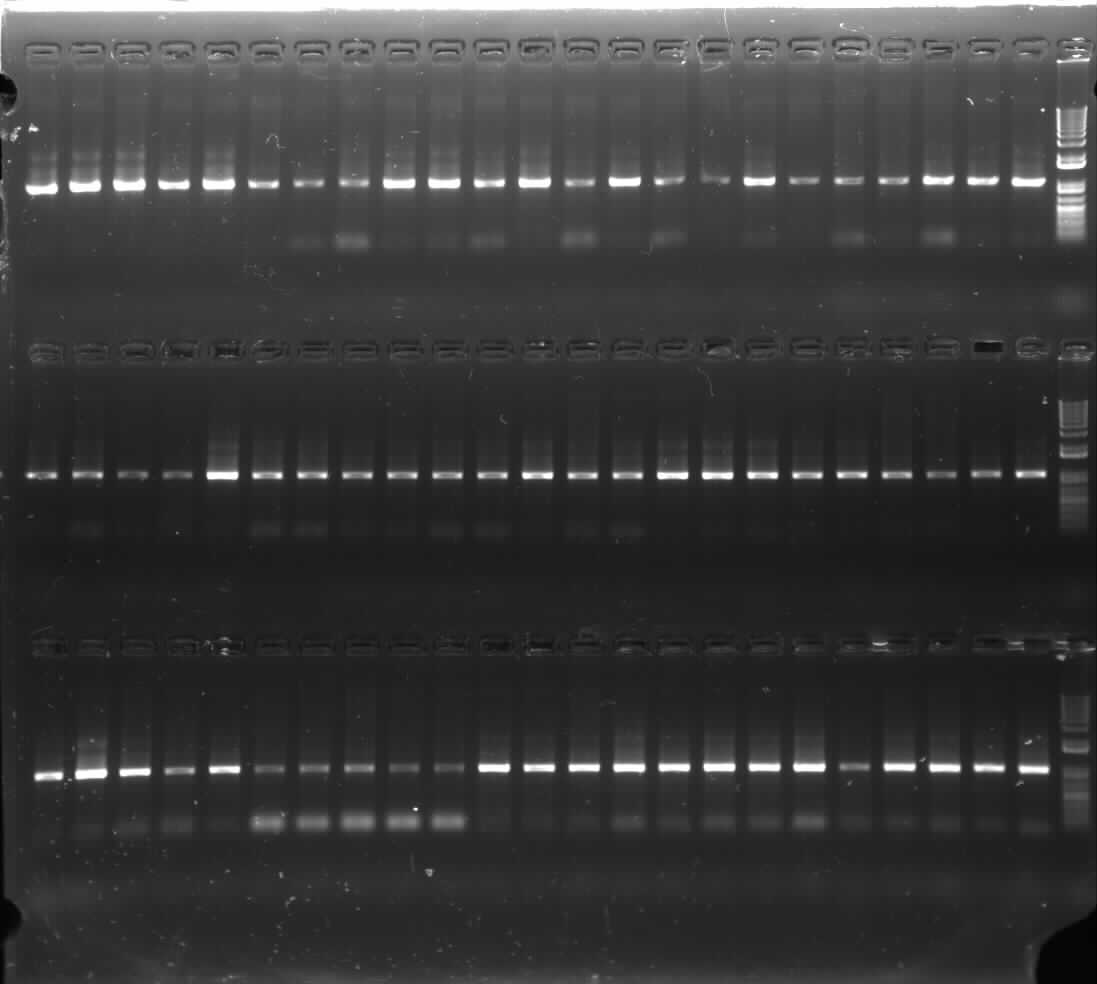


Control: 47 - 69

Control: 24 - 46

Control: 1 - 23


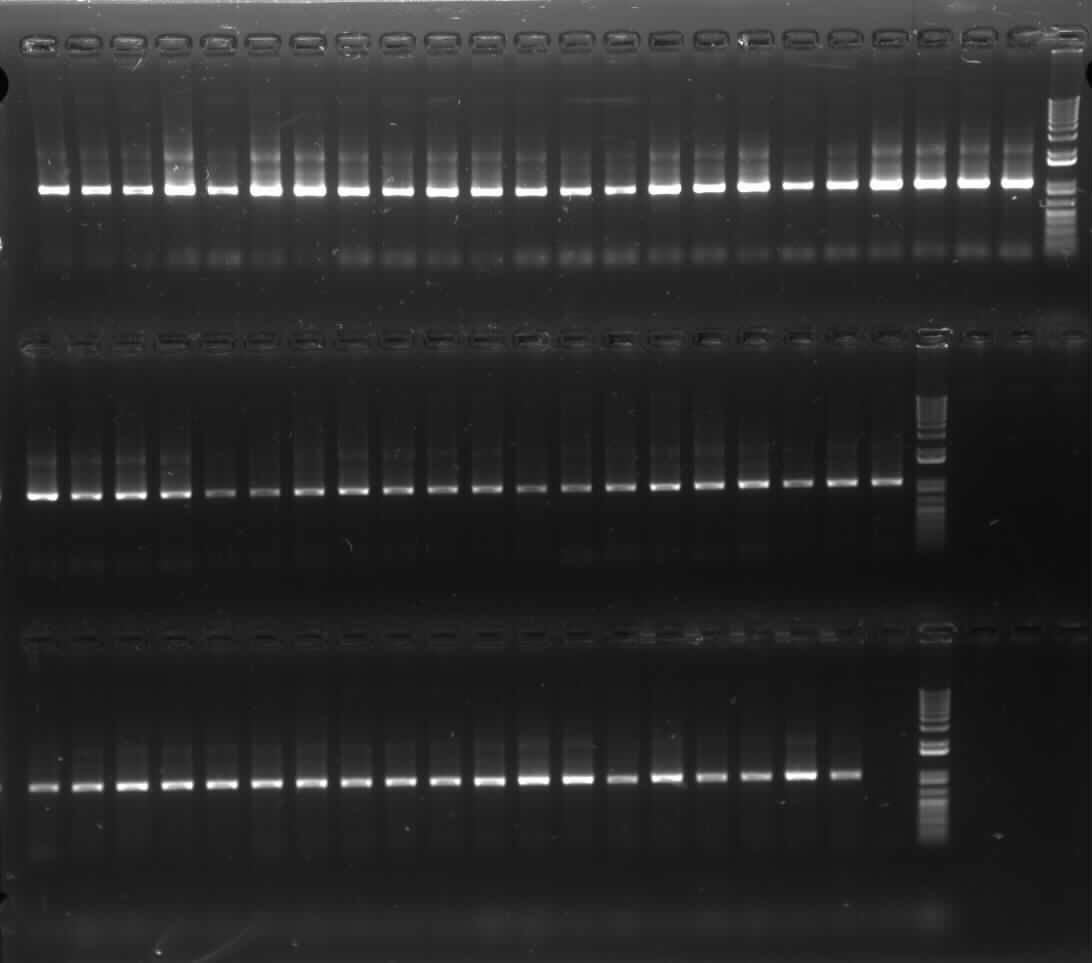


Control: 113 - 131

Control: 93 - 112

Control: 70 - 92

**Figure S3 | Final figure and original gel images of figure S3.** The final figure is shown on the top with the original gel images below with the cropped regions shown as white boxes.

1. **Original gel images of Figure S3**

**
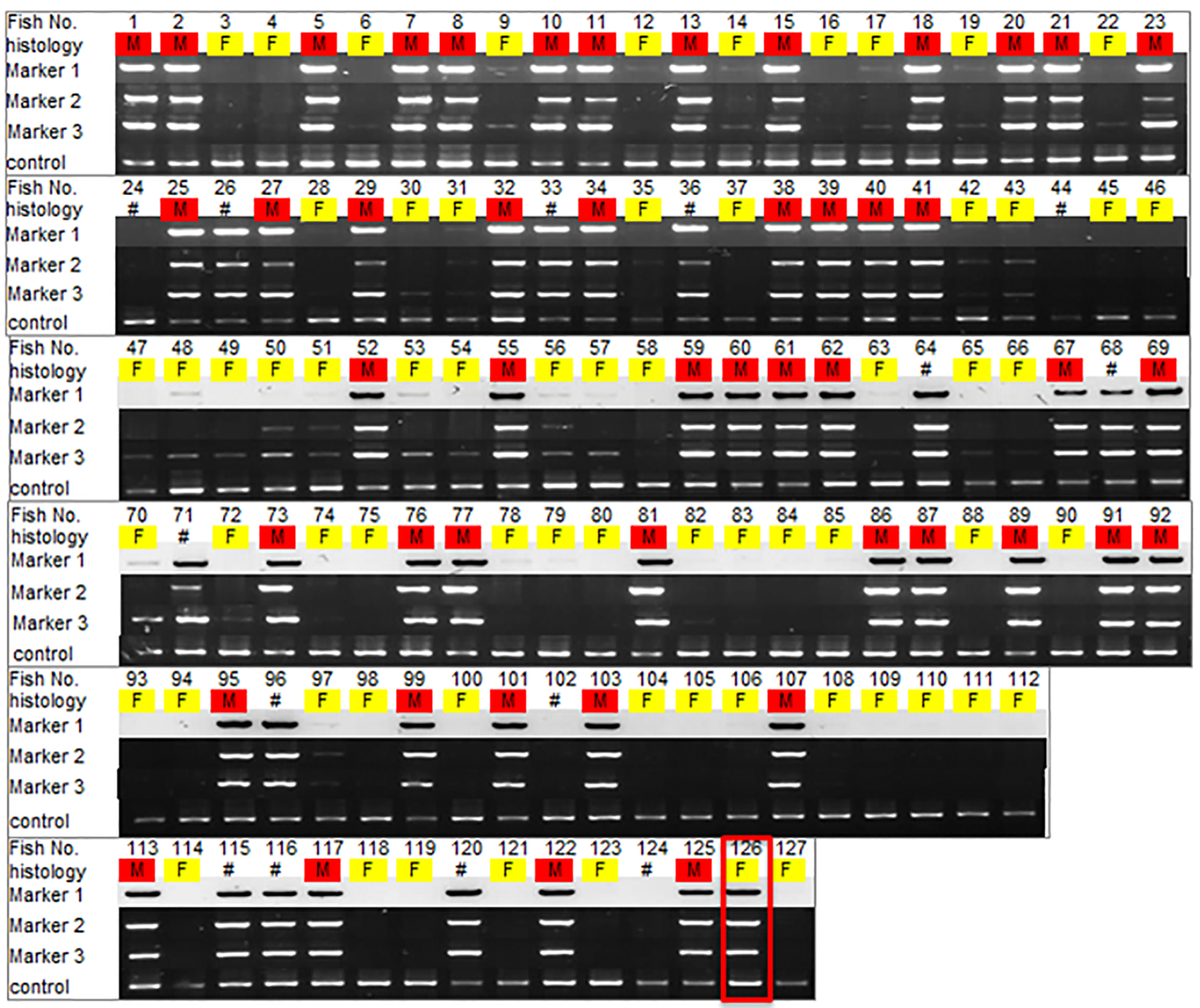
**

Original gel figure for marker 1:


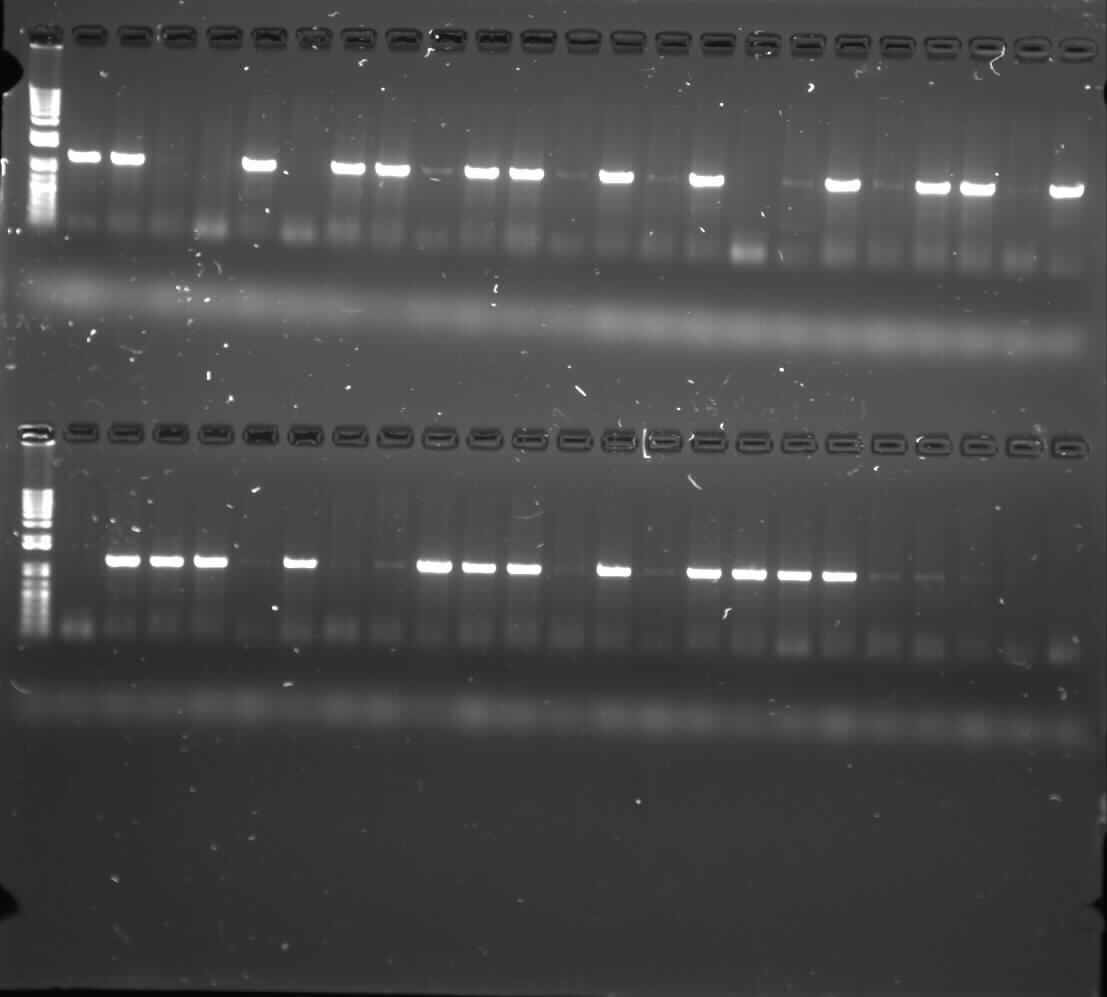


Marker 1: 24 - 46

Marker 1: 1 - 23


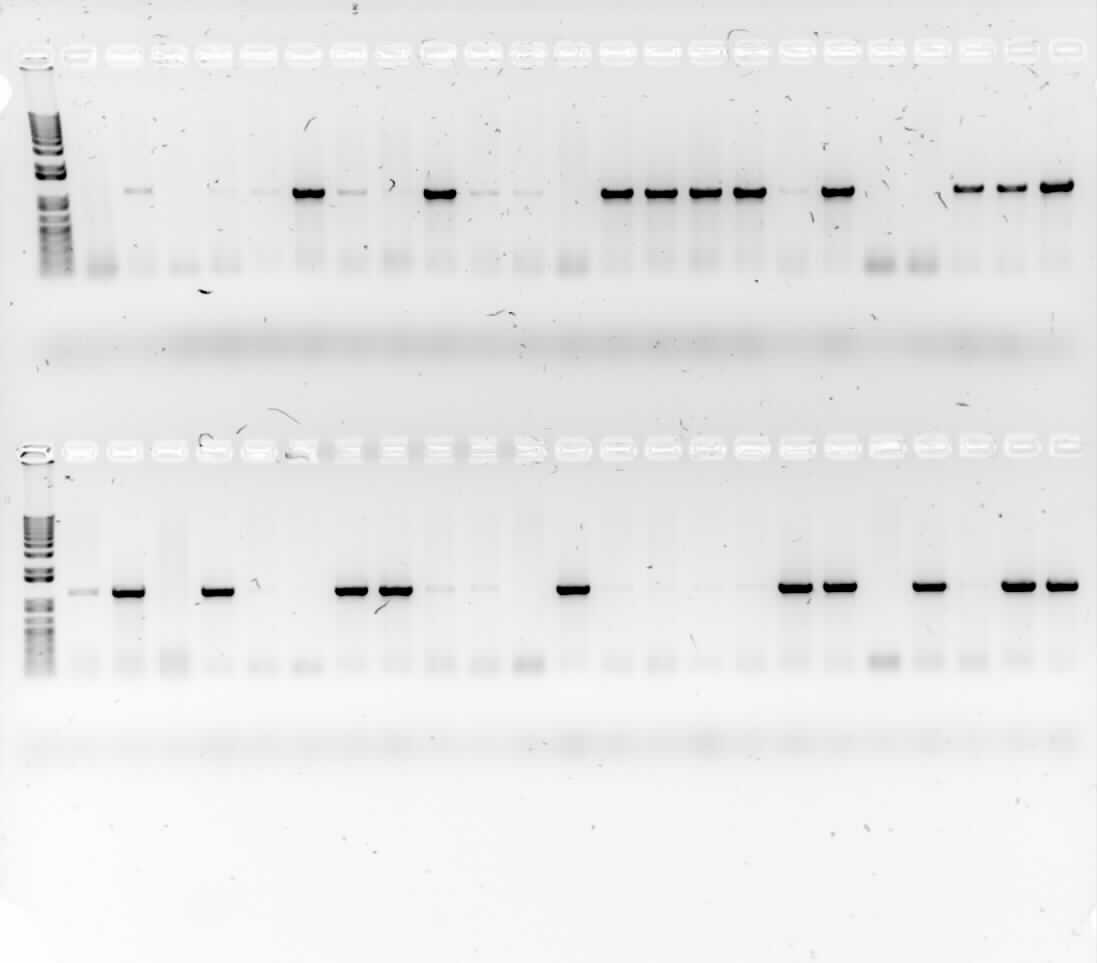


Marker 1: 93 - 112

Marker 1: 47 - 92


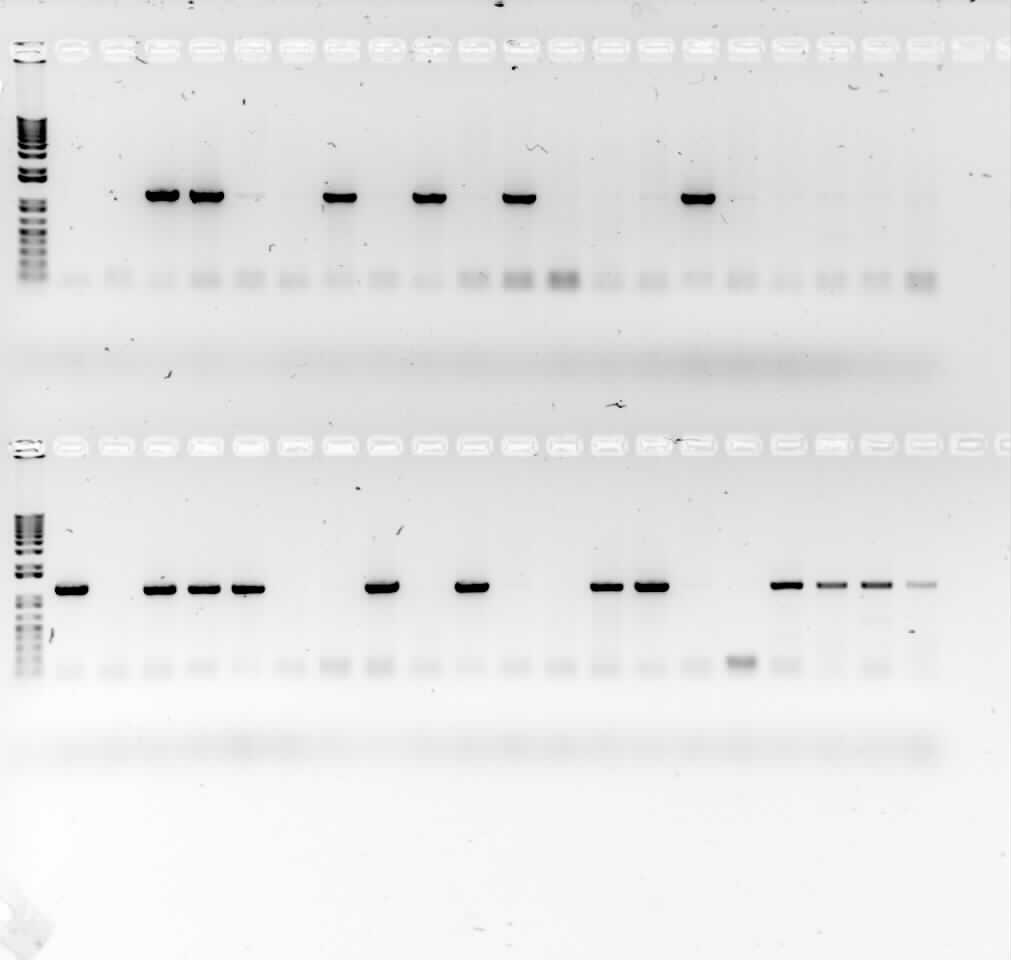


Marker 1: 113 - 127

Marker 1: 93 - 112

Original gel figure for marker 2:


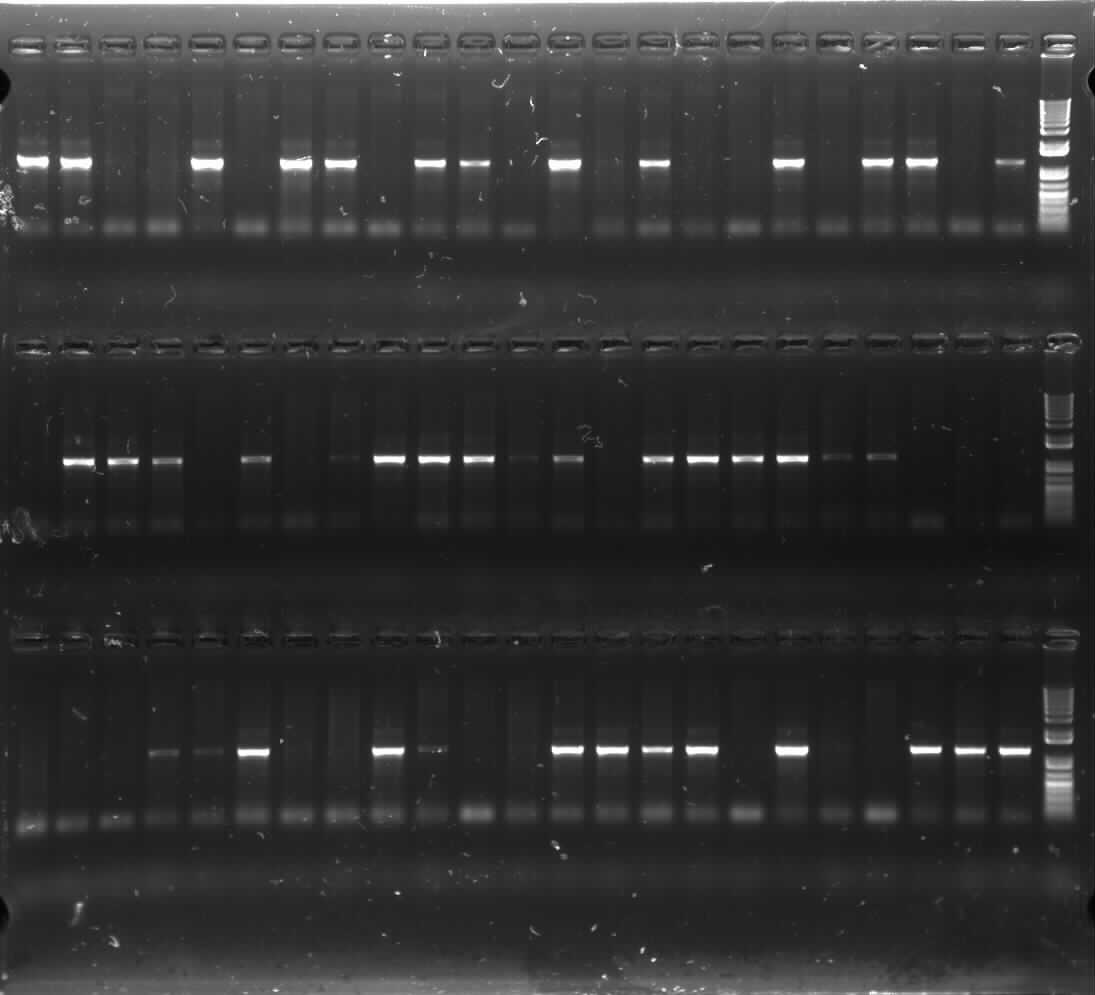


Marker 2: 47 - 69

Marker 2: 24 - 46

Marker 2: 1 - 23


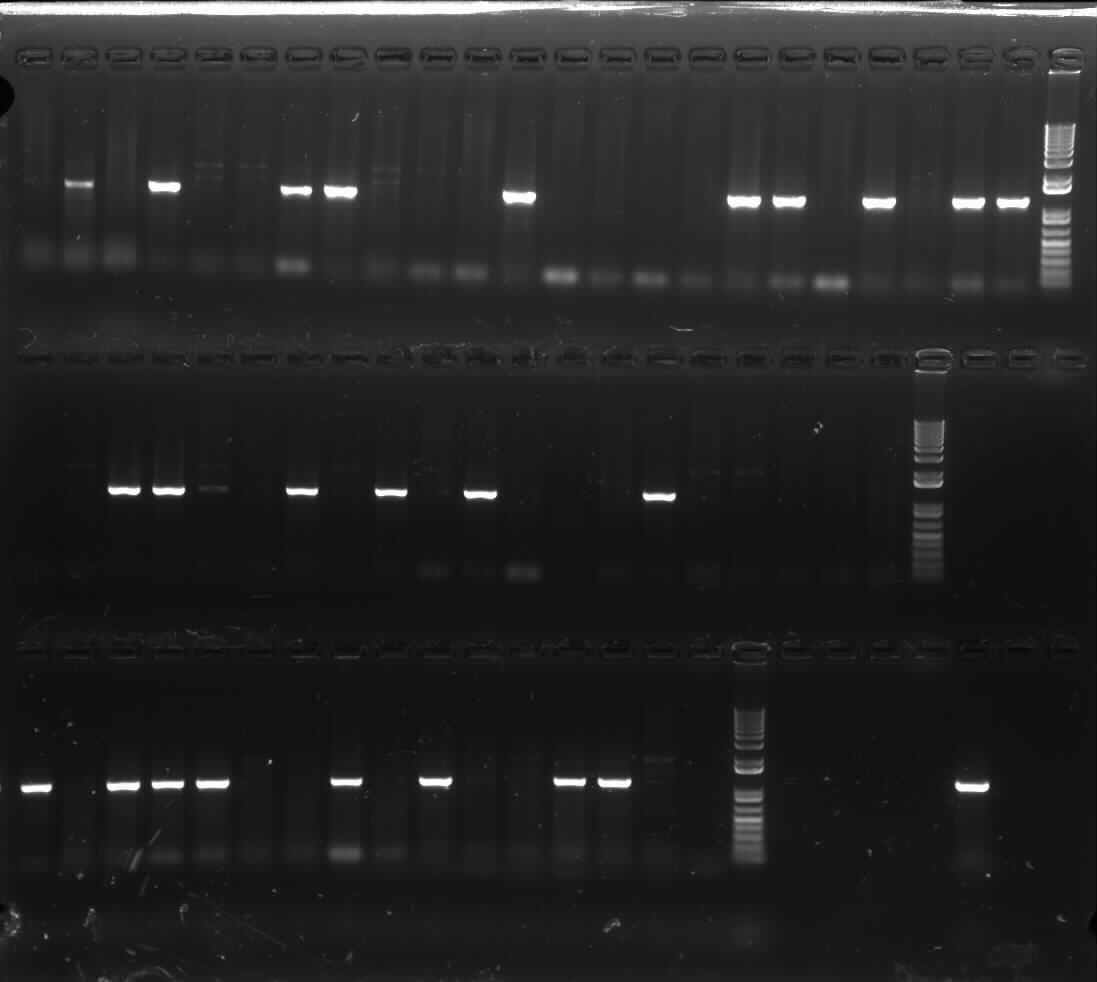


Marker 2: 113 - 127

Marker 2: 93 - 112

Marker 2: 70 - 92

Original gel figure for marker 3:


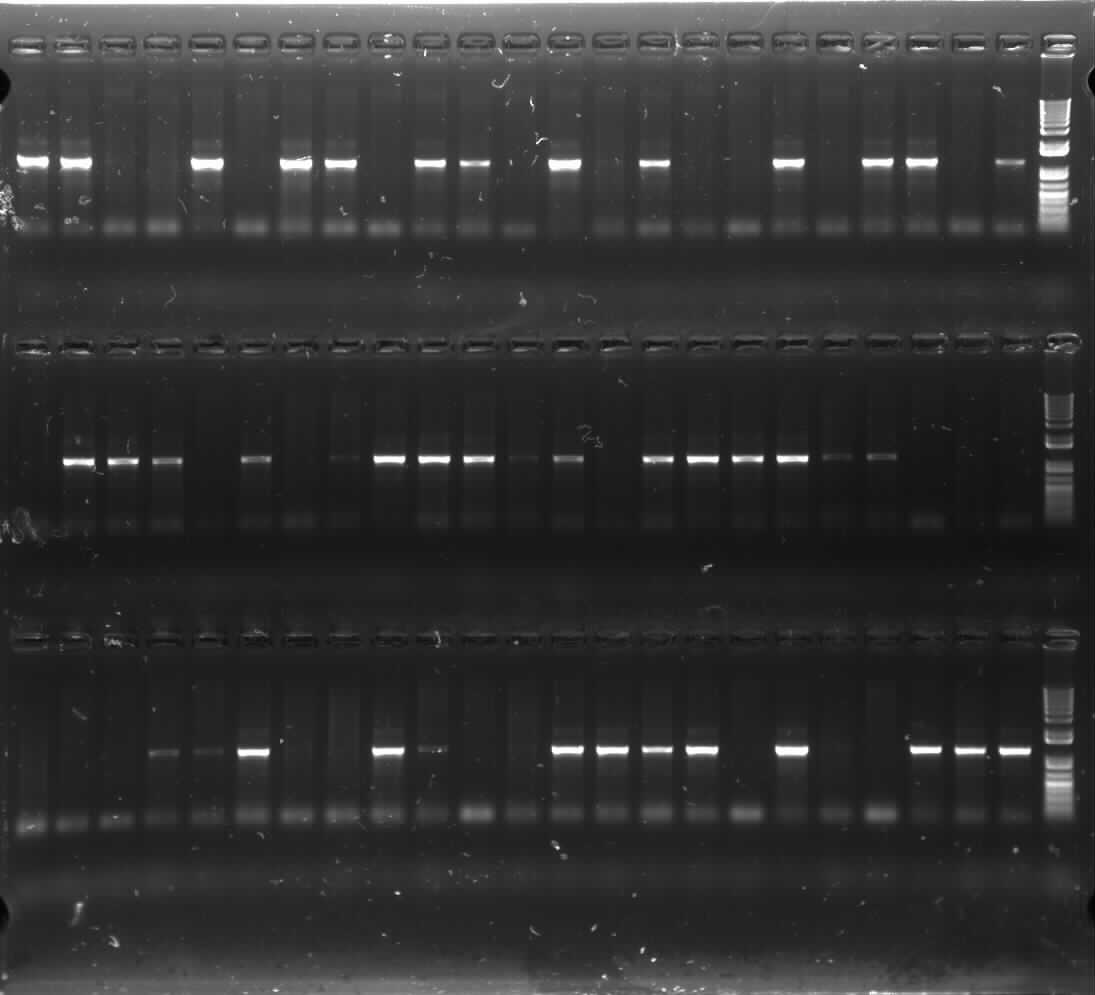


Marker 3: 47 - 69

Marker 3: 24 - 46

Marker 3: 1 - 23


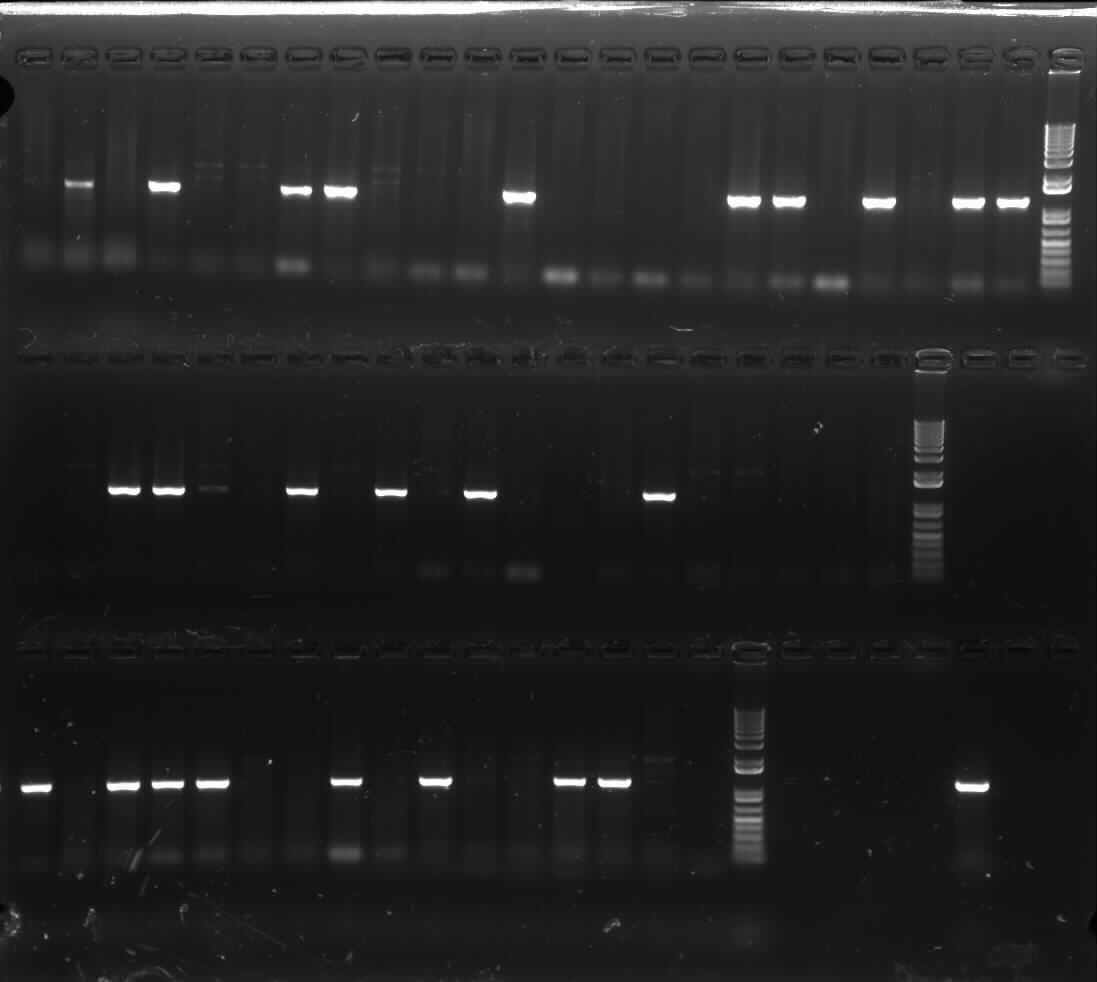


Marker 3: 113 - 127

Marker 3: 93 - 112

Marker 3: 70 - 92

For control:


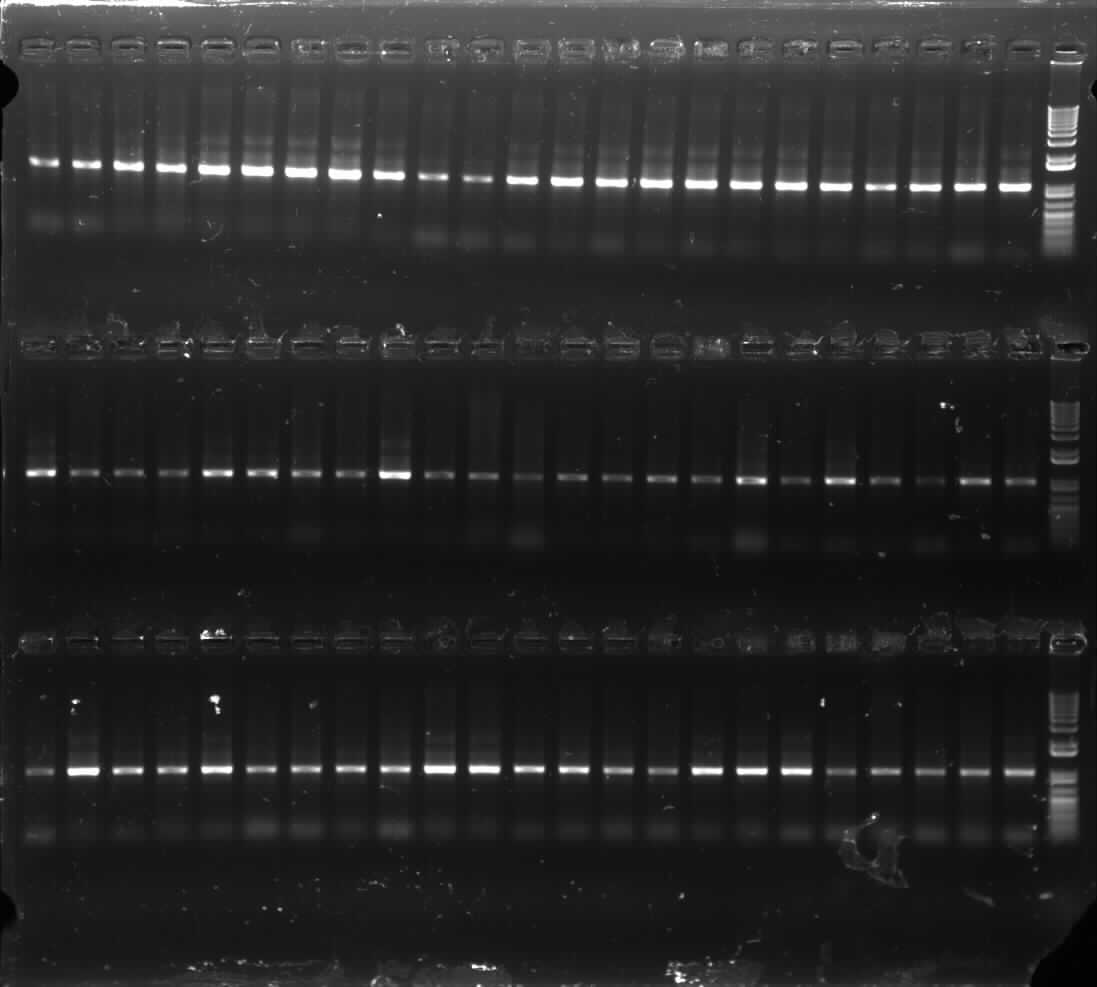


Control: 70 - 92

Control: 24 - 69

Control: 1 - 23


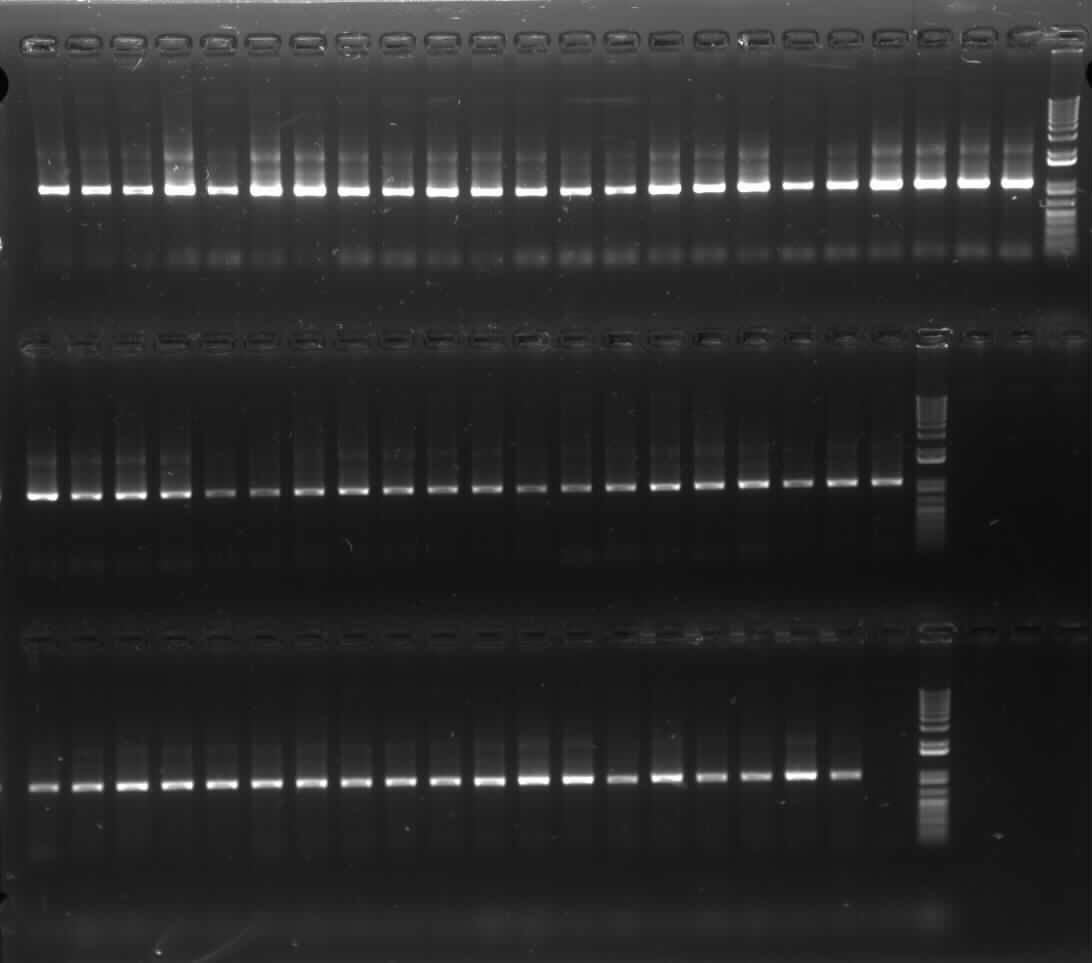


Control: 113 - 127

Control: 93 - 112

Control: 93 - 112

**Figure S4 | Final figure and original gel images of figure S4.** The final figure is shown on the top with the original gel images below with the cropped regions shown as white boxes.
